# Bacterial Community and Spoilage Profiles Shift in Response to Packaging in Yellow-Feather Broiler, a Highly Popular Meat in Asia

**DOI:** 10.3389/fmicb.2017.02588

**Published:** 2017-12-22

**Authors:** Huhu Wang, Xinxiao Zhang, Guangyu Wang, Kun Jia, Xinglian Xu, Guanghong Zhou

**Affiliations:** ^1^National Center of Meat Quality and Safety Control, Nanjing Agricultural University, Nanjing, China; ^2^Jiangsu Collaborative Innovation Center of Meat Production and Processing, Quality and Safety Control, Nanjing Agricultural University, Nanjing, China

**Keywords:** yellow-feathered broiler, metagenomics, bacteria diversity, packaging, spoilage

## Abstract

The consumption of yellow-feathered broiler has been advocated for purchasing with chilled meat rather than live broilers in Asia due to the outbreaks of animal influenza. Here, the microbial community of chilled yellow-feathered broiler response to modified-air packaging (MAP, 80% CO_2_/20% N_2_) and penetrated-air packaging (PAP, air-filling) during storage was revealed by a combination of whole-metagenome shotgun sequencing and traditional isolation methods, and the volatile organic compounds and proteolytic activity of representative dominant isolates were also accessed. The results revealed that MAP prolonged shelf life from 4 to 8 days compared to PAP, when the numbers of total viable counts and lactic acid bacteria reached more than 7 log CFU/g. *Aeromonas*, *Acinetobacter*, *Escherichia*, and *Streptococcus* occupied the bacteria communities in initial broiler carcasses. MAP dramatically increased the bacteria diversity during storage compared to PAP. Clear shifts of the dominant bacteria species were obviously observed, with the top genera of *Aeromonas*, *Lactococcus*, *Serratia*, and *Shewanella* in MAP, whereas the microbial communities in PAP were largely dominated by *Pseudomonas*. The isolates of *Pseudomonas* from PAP carcasses and *Aeromonas* from MAP carcasses displayed strong proteolytic activities. Meanwhile, the principal component analysis based on the volatile organic compounds indicated that the metabolic profiles greatly varied between each treatment, and no link between the natural odor of spoilage meat *in situ* and the volatile odor of the dominant isolates incubated in standard culture was found. These data could lead to new insights into the bacteria communities of yellow-feathered broiler meat during storage and would benefit the development of novel preservative approaches.

## Introduction

The consumption of broilers, which is an indispensable source of meat popular worldwide, has been increasing over the last few years. The yellow-feathered broiler is a traditional poultry breed in Asia that is well known for its unique meat flavor. In 2015, the production (head units) of live yellow-feathered broiler in China has exceeded that of white-feathered broilers such as arbor acres, avian and ross, reaching more than 4.4 billion heads. Traditionally, the live yellow-feathered broilers approved by the purchaser were individually sold and immediately slaughtered in wet markets, and there was usually less than 2 h between live broiler slaughtering and the meat being cooked. Therefore, shelf life was not a noteworthy concern in this process. However, the traditional consumption pattern of yellow-feathered broilers has recently been prohibited by the Chinese government due to outbreaks of animal influenza such as H7N9. Recently, a new consumption pattern of “slaughtered in large-scale plants and sold with chilled meat” has been advocated nationwide and implemented for yellow-feathered broilers. In this pattern, there are usually more than 60 h between live broiler slaughtering and meat cooking. Thus, the microorganism loading on the broiler carcasses is an important concern, and innovative solutions are required to inhibit bacteria growth and extend the shelf life of chilled meat.

Extending the shelf life of chilled yellow-feather broilers is a huge challenge, since the unique flavor is easily lost during storage. Although many approaches have been explored to reduce initial microorganism loading on chicken carcasses and inhibit bacterial growth during storage, such as water-based (hot water, electrolyzed water), chlorine-based, and phosphate-based treatments and organic acids (Loretz et al., 2010; Purnell et al., 2014; Pavic et al., 2015), the drawbacks of these interventions continue to include chlorine residue, discoloration and flavor loss. Therefore, there is an urgent need for the development of novel applications. Previous work has provided evidence that modified-air packaging (MAP) could prolong the shelf life of chilled yellow-feathered meat for more than 6–8 days compared with penetrated-air packaging (PAP) of 2–4 days (Zhang et al., 2015). MAP could also maintain the unique flavor and meat color. However, how to induce the diversity and metabolic profiles shift of microorganisms during storage by the packaging patterns is still unknown. A thorough understanding of the bacterial populations and individual responses to packaging conditions is highly desirable.

Exploring the diversity of microorganisms in foodstuffs and processing environments has become a focus of research, since a better understanding of the role of microbiota in food quality and safety will directly benefit human health (Flores et al., 2013). Extensive surveys have focused on precisely which bacteria occupied the dominant population during the storage of food (Kergourlay et al., 2015; Stoops et al., 2015) and investigated the initial bacterial community of food by culture-independent techniques, such as PCR-DGGE and 16S rDNA amplicon sequencing (Oki et al., 2014; Bozoudi et al., 2016). However, the current research concerns have almost completely focused on the initial food products and ignored the effect of packaging conditions on bacterial communities during storage, and the research techniques used in previous reports based on the partial sequence of bacterial 16S rDNA could not provide a complete picture of the microbial community and lead to ambiguous results due to inherent limitations and errors. Compared to amplicon sequencing, whole-metagenome shotgun (WMS) was another novel metagenomics (Walsh et al., 2017), it was an approach that total genomic DNA extracted from a mixed microbial community is fragmented and sequenced to determine in a non-specific manner the entire gene content of a sample (Franzosa et al., 2015). It could provide better insight into the organization, interaction, evolution, and metabolism of microorganisms. The WMS has exerted a profound impact on our understanding of the ecosystems of environmental microorganisms. However, the use of WMS relying on the whole microbiota in food remains limited.

Therefore, the purpose of this study was to (i) reveal the bacterial community shift of yellow-feather broiler response to packaging patterns during storage, using a combination of WMS and traditional isolation methods, and (ii) access the spoilage profiles, including volatile organic compounds and proteolytic activity, of dominant isolates. This work would lead to a better understanding of diversity, dynamics and metabolic profiles of chilled broiler meat during storage.

## Materials and Methods

### Samples Preparation

**FIGURE 1:**
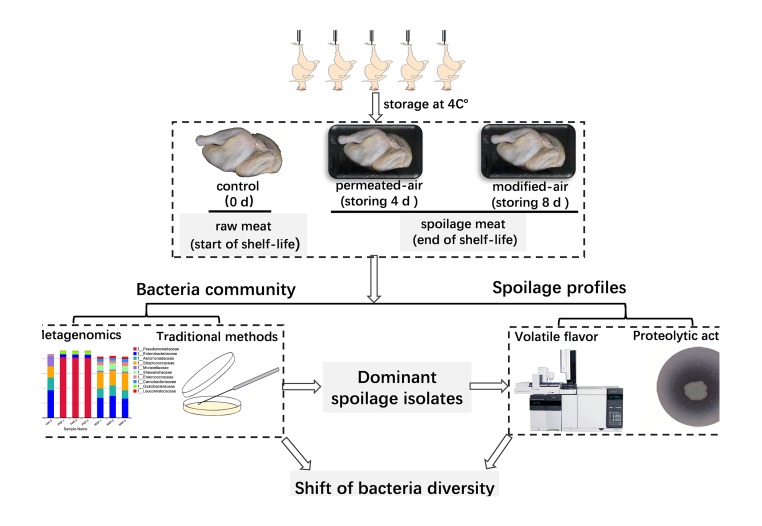
The whole research proposal of this working.

The yellow-feathered broilers were randomly collected from the packaging site of a commercial slaughtering-line in a large-scale plant in China. The carcasses that had not been packaged and stored (0 day) were regarded as control samples. The carcasses were packaged with penetrated-air packaging (PAP, air-filling) or modified-atmospheres packaging (80% CO_2_/20% N_2_) and then were stored at 4°C for 4 and 8 days, respectively. MAP was applied with a polyethylene/polyamide/low-density polyethylene film, with O_2_ permeability of 24 cm^3^/(m^2^⋅day⋅atm), CO_2_ permeability of 78 cm^3^/(m^2^⋅day⋅atm), and water vapor permeability of 44 g/(m^2^⋅day). PAP was applied with a high-density polyethylene film, with O_2_ permeability of 14483 cm^3^/(m^2^⋅day⋅atm), CO_2_ permeability of 63683 cm^3^/(m^2^⋅day⋅atm), and water vapor permeability of 54 g/(m^2^ day⋅atm). The whole research proposal of this study and more detailed information is shown in **Figure 1**.

### Numeration and Collection of Microorganisms

The total microorganisms from broiler carcasses were collected by carcass-washing methods: a prepared half carcass (about 700 g weight) was transferred into a stomacher blender bag containing 300 mL 0.85% NaCl and 0.1% peptone water, and then the sample was shaken for 10 min with 100 rpm. Total viable counts of each sample were determined with plate count agar (PCA, Beijing Land Bridge Technology, Co., Ltd., Beijing, China) according to the China National Food Safety Standard methods (GB 4789.2-2010). Lactic acid bacteria were determined with MRS agar (Oxoid) according to the method proposed by Chouliara et al. (2007). Four independent carcasses were replicated for the numeration of total viable counts and lactic acid bacteria.

The homogenized liquid of each carcass was centrifuged with 1500 ×*g* (10 min, 4°C) to remove various meat tissues, and the supernatant was further filtered with a 0.22 μm pore diameter filter as described by Takahashi et al. (2017). Subsequently, the filter was inserted into a tube containing several beads, and was vortexed and washed three times with 1 mL of 0.85% NaCl, and then microorganisms were gathered by centrifugation with 10,000 ×*g* for 5 min. Total DNA of microorganisms was directly extracted using DNA Microbiome Kit (Qiagen) following the manufacturer’s recommendations. Three replications were performed for DNA extraction and metagenome sequencing.

### Library Construction and Metagenome Sequencing

The integrity, purity and concentration of total DNA was analyzed using 1% agarose gel electrophoresis, NanoPhotometer spectrophotometer (IMPLEN, Westlake Village, CA, United States) and Qubit dsDNA HS Assay Kit in Qubit 2.0 Fluorometer (Life Technologies, Carlsbad, CA, United States). The total DNA of each sample that passed quality control was used for library construction. A total amount of 1 μg DNA per sample was used as input material for the DNA sample preparations. Sequencing libraries were generated using NEBNext Ultra^TM^ DNA Library Prep Kit for Illumina (NEB, United States) following the manufacturer’s recommendations and index codes were added to attribute sequences to each sample. Briefly, the DNA sample (*n* = 3) was fragmented by sonication to a size of 300 bp, and then DNA fragments were end-polished, A-tailed, and ligated with the full-length adaptor for Illumina sequencing with further PCR amplification. At last, the PCR products were purified (AMPure XP system), and the concentrations of amplification were primarily quantified by Qubit2.0, and then the concentrations were diluted to 2 ng/uL. All libraries were analyzed for insert size distribution by Agilent 2100 Bioanalyzer, and finally, the concentration of library (>3 nM) was accurately quantified using real-time PCR to ensure the quality of libraries. The clustering of the index-coded samples was performed on a cBot cluster generation system according to the manufacturer’s instructions. After cluster generation, the library preparations were sequenced on an Illumina HiSeq 4000 platform in Novogene Bioinformatics Technology, Co., Ltd. (Beijing), and the 150 bp paired-end reads were generated.

### Assembly, Annotation, and Taxonomy of Metagenomic Data

The raw reads were filtered by removing low-quality sequences to obtain clean reads according to the following criteria: (1) removing reads containing N base more than 10 bp; (2) removing reads overlapping with adapter more than 15 bp; (3) removing reads with low-quality (quality values ≤ 38) more than 40 bp; and (4) removing reads probably from host by blasting the genome using SOAPAligner software. A total of 56,524.61 Mbp clean data were obtained from Illumina HiSeq system, with an average of 6,280.51 Mbp (Supplementary Table S1), indicating that a deep coverage was achieved, and the detail information of sequencing depth of metagenomics in each sample was shown in Supplementary Figure S1. All the downstream analyses were performed using high-quality clean data. Metagenomic sequence data is available at SRA site from NCBI with SAMN08123132 of biosample identifier (BioProject accession: PRJNA420874; SRA: SRS2729591).

The single-piece and mixture group assembly analysis was performed using SOAPdenovo software, and total of 820,974,610 bp scaffolds and 775,303,201 bp scaftigs were, respectively, obtained (Supplementary Table S1). Based on these assembly scaftigs (> =500 bp), total of 1,171,588 open reading frames (ORFs), with an average of 117,159 ORFs in each sample (Supplementary Table S2), were predicted using the MetaGeneMark software, and following by removing the redundancy of ORFs using CD-HIT software, total of 403,555 gene catalogs (unigenes) were obtained using SOAPAligner software (Supplementary Table S2). The abundances of each unigenes in samples were calculated based on the numbers of reads blasted to unigenes and length of unigenes (Cotillard et al., 2013; Oh et al., 2014). Then each unigene was compared with the NR database in NCBI, and total of 88.48% (357,061) of unigenes could be identified with the criteria of *E* value less than 10^5^, and after filtering the identified unigenes with a standard of minimum *E* value multiply ten (Qin et al., 2010), the annotation of unigenes was carried out using LCA algorithm analysis with MEGAN version, and finally, the taxonomic profiles were obtained based on the abundances and annotations, with 92.57% on family level and 84.00% on genus level, the detail information was shown in Supplementary Table S2.

### Identification of Dominant Isolates

A raw-broiler meat juice agar (RJA) plate prepared by mixing raw chicken juice (the fresh broiler breasts were homogenized with deionized water and then filtered through two layers of gauze) and 1% agar, was used to identify the dominant isolates in PAP and MAP samples. Both ingredients were maintained at 45°C before mixing. RJA plates were sterilized by irradiation at a dose of 6 KGy via the ^60^Co source at Hangyu (Hangyu Irradiation Technology, Co., Ltd., Nanjing, China).

Each 25 g sample of surface meat from spoilage carcasses of PAP and MAP was aseptically weighed and homogenized in 75 mL sterilized 0.85% NaCl solution, and then the solution was streaked on an RJA plate and incubated aerobically at 25°C for 48 h. Each colony was obtained from the plates and then streaked on an RJA plate twice to obtain a single isolate. Total of 23 independent colonies were obtained from PAP samples, and 21 colonies were obtained from MAP samples. The obtained isolates were identified by 16S rRNA gene sequencing. Amplification of the 16S rRNA gene was performed using region-specific primers 27F (5′-aga gtt tga tcc tgg ctc ag-3′) and 1492R (5′-ggt tac ctt gtt acg act t-3′). The amplification products were purified and sequenced by Invitrogen (Invitrogen Biotechnology, Co., Ltd., Shanghai, China). The identified isolates were further confirmed using a VITEK 2 automated system (BioMerieux, France).

### GC–MS Analysis

Four grams meat showing typical spoilage features and a 4 mL supernatant of each isolate identified by a combination of 16S DNA sequencing and VITEK 2 (*Pseudomonas fragi* and *Pseudomonas fluorescens* from PAP meat, *Aeromonas salmonicida* and *Serratia liquefaciens* from MAP meat) incubated for 48 h at 25°C was tested for volatile organic compounds by GC–MS. The approach of supernatant collection was performed as previous reported (Beristain-Bauza et al., 2016). Each sample was performed on an Agilent 7890A gas chromatograph coupled to an Agilent 5973C mass spectrometer. A slight modification of the method described by Jaffres et al. (2011) was used. Briefly, a 4 g or 4 mL portion was transferred into a 20-mL vial with a polypropylene screw-on cap and a PTFE/silicone septum to make it airtight. The vial was heated at 40°C for 40 min to equilibrate the system. The SPME fiber, an 85-μm Carboxen/polydimethylsiloxane StableFlex^TM^ (Supelco), was inserted through the septum and exposed in the headspace of the vial for 30 min to allow absorption of the volatile compounds into the fiber. The SPME was then introduced into the injector port of the gas chromatograph for 5 min in splitless mode, set at 250°C, to desorb the volatile compounds. The desorbed components were analyzed on an Agilent DB-WAX (30 m × 0.25 mm × 0.25 μm) capillary column. Helium was used as a carrier gas at a constant flow rate of 0.8 mL/min, and the oven temperature was programmed as follows: 40°C for 5 min, ramped at 3°C/min until reaching 140°C, ramped at 10°C/min until reaching 250°C, and then held for 5 min. The mass spectrometer was operated in the electron impact mode with the electron energy set at 70 eV and a scan range of 50–500 m/z. The NIST library and comparison with spectra and retention times of the standards were used to identify the volatile components.

### Proteolytic Activity of Isolates

All identified isolates were cultured for 24 h at 25°C. Cell density was adjusted to an optical density of 600 nm of 0.4 value. Two microliter aliquots of isolates were spotted onto an RJA plate. Proteolytic decomposition was measured after 3 days of incubation at 25°C. Four independent replications were performed for each isolate.

### Statistical Analysis

The results of numeration for total viable counts and lactic acid bacteria and the decomposition zone diameters were expressed as the mean ± standard deviation. Statistical significance was determined by a one-way Duncan’s ANOVA procedure using SPSS 13.0. The level of statistical significance was *p* < 0.05.

Principal component analysis (PCA) was used to dissociate the patterns of volatile organic compounds between each packaging pattern. Additionally, PCA was used to determine the relationship between the volatile organic compounds of spoilage meat *in situ* and the volatile organic compounds of isolates incubated in standard TSB. The PCA analysis was conducted by SIMCA-P software (Umetri AB, Sweden).

## Results

### MAP Prolonged the Shelf Life from 4 to 8 Days

**FIGURE 2:**
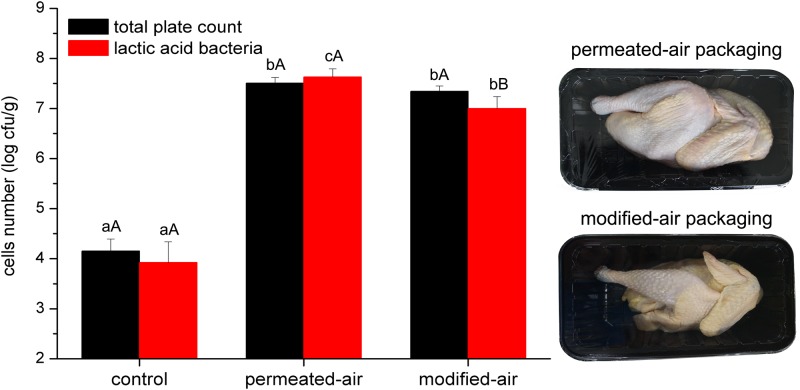
Numbers of bacteria (obtained from plate counting method) of broiler carcass. Error bars represent standard deviations of the mean (*n* = 4). Different lowercase letters at the same indicators and different capital letters at the same treatment are statistically different (*p* < 0.05).

As shown in **Figure 2**, The initial number of bacteria was approximately 4.0 log CFU/g, indicating good hygienic operation of the slaughter plant, which was in line with the production requirements of chilled broiler carcasses. The numbers of total plate count and lactic acid bacteria on carcasses exceeded 7.0 log CFU/g after storage for 4 and 8 days for PAP and MAP, respectively, which was widely considered as the threshold of microorganism counts during meat spoilage. No difference in the numbers of both kinds of bacteria between PAP and MAP was found at the end of shelf life. Compared to PAP, MAP prolonged the shelf life by 4 days.

### MAP Obviously Increased the Diversity of Bacteria and Altered the Top Species of Spoilage Bacteria

**FIGURE 3:**
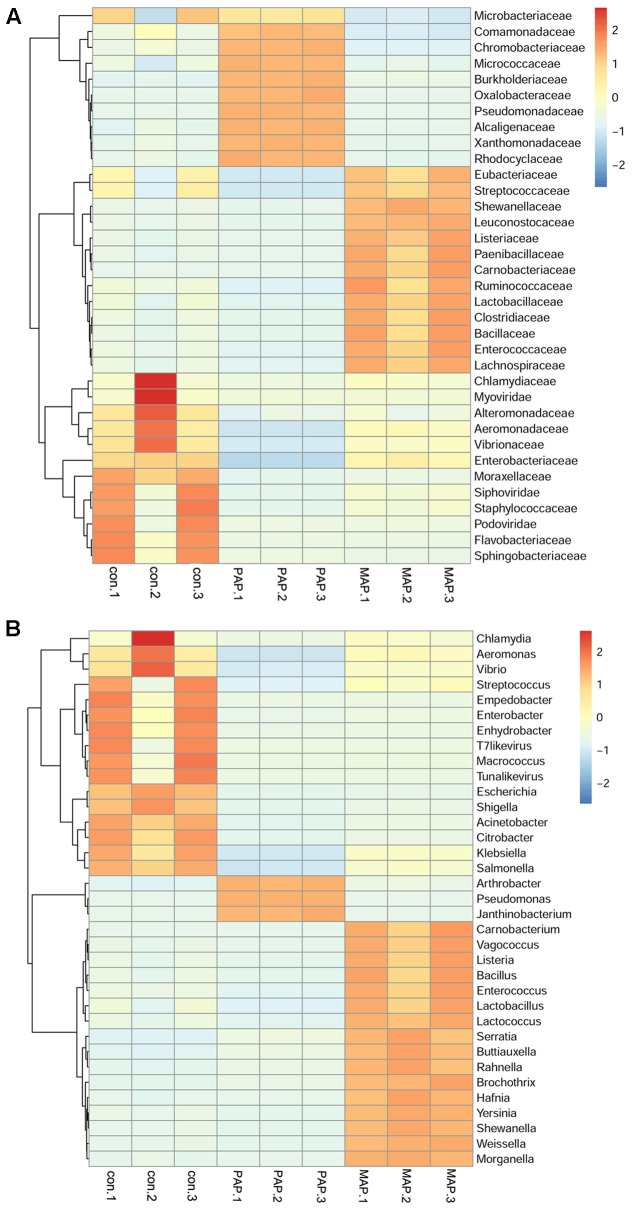
The heat map of relative abundance within each sample of the top 35 bacterial in family **(A)** level and genus **(B)** level.

The sequence statistics and data summary are shown in **Table 1**. The sufficient amount and high quality of clean data could guarantee the accuracy of metagenomics. The clean data were used to determine diversity analysis. The heat map shown in **Figure 3** revealed the dynamics of the 35 most abundant bacteria along with the packaging patterns. The packaging patterns greatly influenced the bacterial diversity of broiler carcasses at the family level and genus level. The dominant bacteria genera in control samples mainly included *Streptococcus*, *Enterobacter*, *Empedobacter*, *Macrococcus*, *Enhydrobacter*, and *Aeromonas*. Compared with control treatment, PAP caused a shift in microorganism diversity, with an increase in abundance of *Pseudomonas*, *Arthrobacter*, and *Janthinobacterium* and a decrease in *Aeromonas*, *Citrobacter*, and *Klebsiella*. In contrast, MAP dramatically increased the relative abundance of *Carnobacterium*, *Vagococcus*, *Enterococcus*, *Lactobacillus* and *Serratia*, as well as *Hafnia*. *Shewanella* and *Weissella*, which are commonly associated with food spoilage, showed lower abundance in both control and PAP treatments. Unexpectedly, high abundances of several food pathogens (**Figure 3**), such as *Salmonella*, *Shigella*, *Vibrio* and *Yersinia*, were observed in all groups, especially for control samples, indicating that more effective interventions should be applied to control pathogens in the slaughter plant.

**Table 1:** Sequence statistics and data summary for the tested broilers.

Sample	Insert size (bp)	Strategy	Raw data (Mbp)	Clean data (Mbp)	Q20	Q30	Effective (%)
Con.1	300	(150:150)	6264.86	6253.03	93.77	86.27	99.811
Con.2	300	(150:150)	7194.72	7158.76	94.64	88.52	99.5
Con.3	300	(150:150)	6643.06	6630.16	94.77	87.98	99.806
PAP.1	300	(150:150)	6992.34	6988.68	93.72	85.71	99.948
PAP.2	300	(150:150)	5516.16	5509.77	94.15	86.72	99.884
PAP.3	300	(150:150)	5995.02	5988.58	93.04	84.76	99.893
MAP.1	300	(150:150)	5928.04	5916.40	94.32	87.31	99.804
MAP.2	300	(150:150)	6771.33	6752.41	95.66	89.91	99.721
MAP.3	300	(150:150)	5337.15	5326.82	94.56	87.66	99.807

*PAP, penetrated-air packaging filled with air; MAP, modified-air packaging filled with 80% CO_2_ and 20% N_2_.*

**FIGURE 4:**
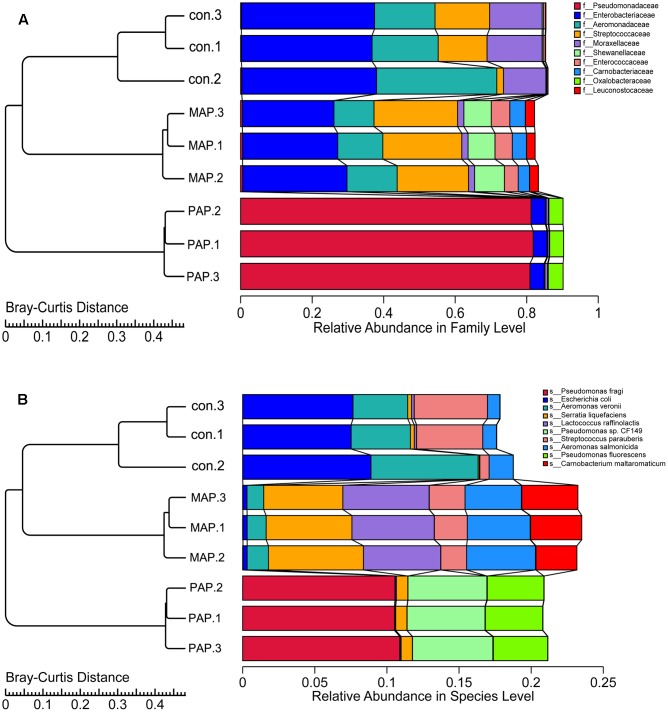
Clustering tree based on the Bray–Curtis distance obtained from the relative abundance in family **(A)** and species **(B)** levels of all samples.

**FIGURE 5:**
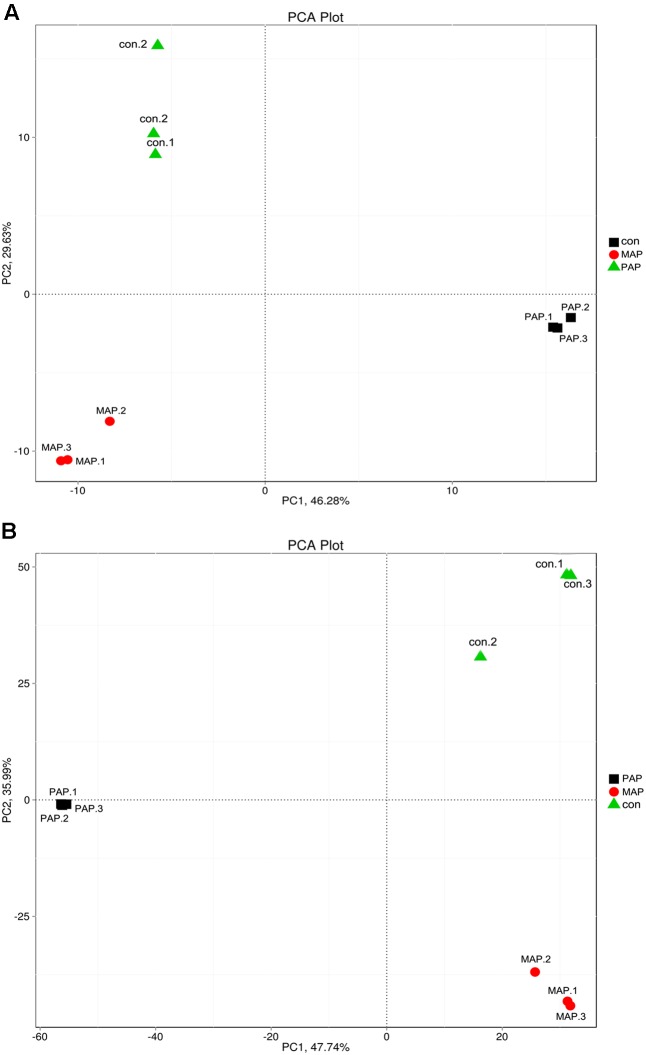
Principal component analysis (PCA) based on the bacteria diversity in family **(A)** and species **(B)** levels of all samples.

The clustering tree based on the Bray–Curtis distance obtained from the relative abundances in family and species levels is shown in **Figure 4**. The results indicated that a high similarity in relative abundance was found between each duplicate in individual treatments, and low similarity was found between each treatment, directly suggesting that the repeatability of microorganism diversity within the same treatment was fairly good. Additionally, the dynamic of bacterial diversity was dramatically influenced, in particular at the species levels, which showed that MAP significantly increased the diversity of bacteria and altered the dominant bacterial species. The results mentioned above indicated that the bacterial community shift was greatly influenced by packaging patterns, and this finding was consistent with the results reported in **Figure 5**, which come from the PCA based on the bacterial diversity at the family and species levels.

**FIGURE 6:**
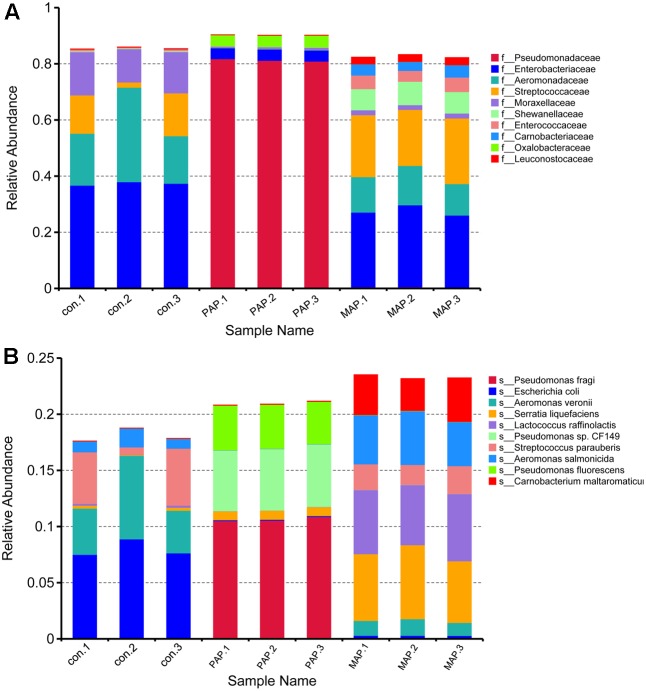
Top 10 bacteria obtained from the relative abundance in genus **(A)** and species **(B)** levels.

Additionally, the structure of the top 10 dominant bacteria at genus and species levels was dramatically affected by the packaging patterns (**Figure 6**). Compared to control treatment, the abundances of *Escherichia coli* and *Aeromonas veronii* were dramatically reduced in MAP and PAP treatments, whereas the abundances of *Lactococcus raffinolactis* and *Pseudomonas* sp. (including *fragi*, *CF149*, and *fluorescens*) were sharply increased in MAP and PAP, respectively, especially for *Pseudomonas* sp., which occupied a dominant abundance in PAP at the end of shelf life. In contrast, *Serratia liquefaciens*, *L. raffinolactis*, *Aeromonas salmonicida*, and *Carnobacterium maltaromaticum* together become the dominant bacteria in MAP at the end of shelf life.

The plate streaking methods (RJA plates) was used to confirm the species of dominant spoilage bacteria. The bacteria of *P. fragi* (eight isolates), *P. fluorescens* (nine isolates) and *C. shigense* (six isolates), were identified in PAP samples, and the isolates of *A. salmonicida* (eight isolates), *A. hydrophila* (five isolates), *A. media* (three isolates), and *S. liquefaciens* (five isolates) were identified in MAP samples.

### Packaging Altered the Metabolic Profiles of Bacteria Grown on Meat *in Situ*

**FIGURE 7:**
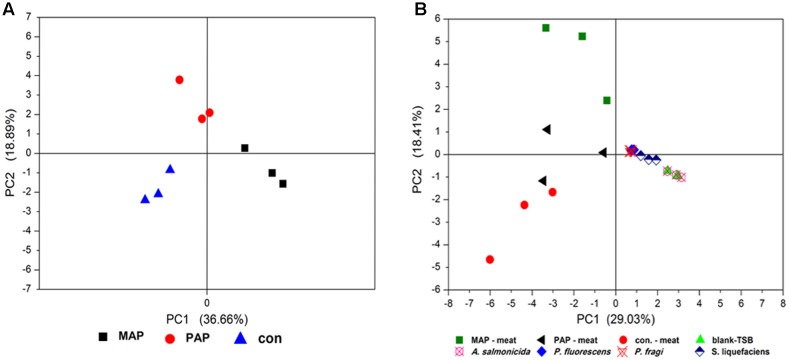
Principal component analysis based on the volatile organic compounds analysis of spoilage meat *in situ* **(A)** and a combination of spoilage meat *in situ* and the supernatant of isolates incubated in standard culture **(B)**.

To explore the metabolic profiles of microorganisms subjected to packaging patterns, the volatile organic compounds of broiler meat under corresponding packaging at the end of shelf life were investigated. All compounds were further analyzed by PCA. Packaging patterns significantly altered the volatile organic compounds of meat (**Figure 7A**), which were mainly associated with the metabolic profiles of spoilage bacteria. To explore the relationship between the metabolic profiles of dominant spoilage isolates and bacteria community subjected to packaging, the PCA based on the volatile organic compounds of spoilage meat *in situ* and the supernatant of identified isolates incubated by GC–MS was determined (**Figure 7B**). Great differences in volatile organic compounds between *A. salmonicida* isolates and the other three isolates were observed. No difference in volatile organic compounds was found among *P. fragi*, *P. fluorescens*, and *S. liquefaciens*. Unfortunately, no relationship was determined between the volatile organic compounds of meat *in situ* and the volatile organic compounds from the supernatants of *A. salmonicida*, *P. fragi*, *P. fluorescens*, and *S. liquefaciens*. This finding suggests that the metabolic profiles of spoilage bacteria grown in standard culture could not represent the spoilage potential of dominant bacteria grown in the meat matrix.

### *P. fragi* Showed the Largest Spoilage Activity in Broiler Meat

**FIGURE 8:**
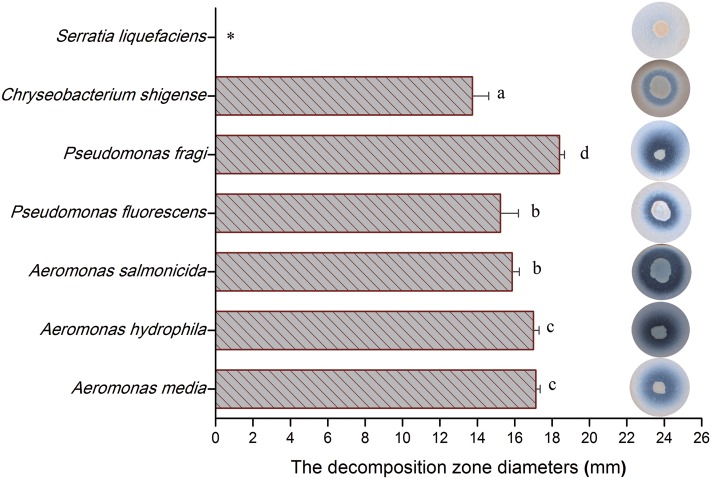
The decomposition zone diameters of identified isolates incubated on raw-chicken juice agar (RJA) plate. Error bars represent standard deviations of the mean (*n* = 4), mean values in column with different lower-case letters are statistically different (*p* < 0.05).

To evaluate the potential spoilage ability of isolates, the identified isolates were individually assessed with decomposition zone diameters. **Figure 8** indicates that there were great differences in proteolytic activity among these tested isolates, with *Pseudomonas fragi* showing the largest proteolytic activity, reaching more than 18 mm in decomposition zone diameter. On the other hand, *S. liquefaciens* had hardly any proteolytic activity in broiler meat. Other isolates showed comparatively strong proteolytic activity, with decomposition zone diameters ranging from 13.5 to 17 mm.

## Discussion

Whole-metagenome shotgun sequencing has proven to be a powerful approach to exploring a large variety of natural microorganisms. WMS could reveal the impact of a given change on the microbiota and their roles of a food system, which is directly correlated to food shelf life, flavor, and other aspects. A combination of WMS and other approaches have been successfully applied to characterize *in situ* microbial diversity and *in vitro* community reconstruction of cheese rind, providing insights into the species and role of specific microorganisms in flavor production during cheese fermentation (Wolfe et al., 2014). The potential roles for *Enterobacteriaceae* and acetic acid bacteria during fermentation process of cocoa bean have been revealed using WMS method (Illeghems et al., 2015). The WMS could be also used to help reduce food spoilage and assist in the optimization of food preservative process (Kergourlay et al., 2015), the applications of WMS has currently affordable not only for scientific researchers but also for the food industry, in particular of revealing the microbial communities and their functional roles involved in traditional foods. In the present study, the traditional broiler carcasses packaged by PAP and MAP were evaluated using WMS approach. It was imperative to explore the diversity and metabolic profiles of microorganisms associated with spoilage and to establish a relationship between microorganism metabolism and the characterization of meat spoilage.

Although MAP has been applied in a wide variety of food products, such as fresh and processed meat, fruit and vegetables, and seafood, the gas composition needs to be optimized to maintain the special eating quality of the corresponding food. Previous finding indicated that a composition of 80% CO_2_/20% N_2_ was suitable for preservation of yellow-feathered broiler carcasses (Zhang et al., 2015), and in the present study, MAP extended the shelf life from 4 to 8 days compared to PAP. More importantly, the diversity of microorganisms and the dominant species were greatly altered by MAP, which may be a potential explanation for extension of shelf life. The initial (control group) dominant bacterial species on carcasses mainly consist of *Aeromonas*, *Acinetobacter*, *Escherichia* and *Streptococcus*, which were commonly present in the hygiene surveys of fresh food and contacted-surface in plants (Manna et al., 2013; Li et al., 2016). An investigation in central India showed that the overall prevalence of *Aeromonas* spp. in various meats from slaughter plants was up to 22% (Gowda et al., 2015). Meanwhile, Arslan and Kucuksari (2015) demonstrated that more than 95% of *Aeromonas* strains showed lipase activity and proteinase activity, and 30% of the strains could produce slime. These characterizations were commonly associated with food spoilage, and the strong proteinase activity of *Aeromonas* isolate was also confirmed in the present study. The types of off-odors, including sour, sulfur, and amine, have been identified in cooked tropical shrimp inoculated with *Aeromonas* followed by storage under MAP (Mace et al., 2014), which was in agreement with the odor characteristic of spoiled broiler carcasses under MAP in the present study.

*Acinetobacter* was another genus with high abundance in the control sample of this study, which dramatically decreased in PAP and MAP treatments. Recent finding has demonstrated that *Acinetobacter* was one of the dominant microbial species in foodservice environments, such as the pre-processing surface, storage room, and kitchen (Stellato et al., 2015), and this may explain why high abundance of *Acinetobacter* was observed in control samples. Consistent with the finding in this study, little or no abundance of *Acinetobacter* was also identified in meat and aquatic products under aerobic and CO_2_/N_2_ modified atmospheres during cold storage in previous studies (Coton et al., 2013; Holl et al., 2016). Conversely, Zhang et al. (2012) found that *Acinetobacter* was always the dominant species in air-packaged broiler during storage. Differences in applied experimental conditions (such as packaging film properties, diversity of dominant species in initial samples) could be a possible explanation for such a discrepancy. *Escherichia* and *Streptococcus* were also identified as the dominant species initially present in broiler carcass. Although the abundance of *Streptococcus* decreased after storage, a certain abundance of *Streptococcus* was still present in the MAP treatment. Consistent results were found by Fernandez-No et al. (2012), who first identified two isolates of *Streptococcus parauberis* in seafood product that was spoiled vacuum-packaged and refrigerated.

*Pseudomonas* becomes the absolute dominant bacteria genus following PAP, whereas a low abundance of *Pseudomonas* was observed in control and MAP groups. It is now commonly accepted that *Pseudomonas* was the dominant species driving the aerobic spoilage of meat products, and different molecular types of *Pseudomonas fragi* have the same overall behavior as meat spoilers (Ercolini et al., 2010). The isolates of *Pseudomonas* in this study showed great proteinase activity, which directly displayed the spoilage characterization of *Pseudomonas*. According to the classical theory of food spoilage assessment (Parlapani et al., 2015), the growth and metabolite of bacteria during food spoilage should: (1) be initially absent or at least present at low abundance levels in food, (2) be driven by the dominant spoilage microorganisms, (3) significantly increase during the storage period, and (4) display good correlation with the growth of microorganisms and score of sensory evaluation. The dynamics and characterization of *Pseudomonas* in the PAP treatment completely conformed to these principles mentioned above. Meanwhile, the absence of *Pseudomonas* following MAP treatment was also consistent with other studies, since the lack of oxygen in the MAP environment greatly prohibited the survival and growth of *Pseudomonas*. Interestingly, the *Serratia* and *Enterococcus* genera were present in all three treatments (control, PAP, and MAP) with high abundances in this study. In particular, in the MAP group, *Serratia* became the dominant bacteria during storage, and a similar finding was recently reported by Holl et al. (2016), who observed that *Serratia* sp. carried out by MALDI–TOF MS was the dominant spoilage microbiota of poultry meat after 8 days at 4°C in MAP (N_2_/CO_2_, 35%/65%). No proteinase activity was observed in *Serratia liquefaciens* isolate in this study, suggesting that the *Serratia liquefaciens* may not contribute to texture softening but off-odor, and this hypothesis was supported by the finding that off-odors of *Serratia* strains from spoiled salmon were recognized as “pyrrolidine” (Mace et al., 2013). *Enterococcus* spp., predominantly present in the gastrointestinal tract of humans and animals, were traditionally isolated from fermented food products, but high occurrence of *Enterococcus* spp. in fresh food have been recently investigated (van Hoek et al., 2015). The human health risks associated with the occurrence of *Enterococcus* in poultry meat has been confirmed (Bortolaia et al., 2016). In addition, a recent study demonstrated that *Enterococci* could be an indicator of potential growth of *Salmonella* in fresh minced meat at retail (Hansen et al., 2016). The abundance of *Enterococcus* was present in the MAP group in this study, indicating that the potential higher risk of broiler consumption-related *Enterococci* species present in the food chain merits further study.

Interestingly, the abundance of *Lactococcus*, *Shewanella*, and *Carnobacterium* genera significantly increased under MAP and finally became the dominant bacteria at the end of shelf life. These species may have come from the food contact surfaces in the broiler slaughter plant via cross-contamination. A similar finding was observed by Rahkila et al. (2012), who isolated several *L. raffinolactis* from skin-containing broiler products under MAP. Meanwhile, other studies have also demonstrated that *Carnobacterium* could grow to be the dominant bacteria in meat products under MAP conditions (Mace et al., 2014). Generally, *Shewanella* was regarded as one of the dominant species of spoiled meat under the air packaging condition. However, a great abundance of *Shewanella* was observed in MAP rather than PAP in the present study. In line with our finding, several *Shewanella* isolates with spoilage characterizations of acid, sour, and amine have been obtained from the spoiled raw salmon stored under modified atmosphere packaging (Mace et al., 2013). Unexpectedly, *Lactobacillus* was not one of the top 10 abundant bacteria in the MAP group, which is typically observed during the spoilage of meat under anaerobic packaging. The discrepancy could partly be attributed to microbial interactions of different test systems.

Off-odor originating from microorganism metabolism was an indispensable index during the evaluation of meat spoilage. The characterization of volatile organic compounds of the present spoilage species has been widely observed in standard growth cultures, which ignore microbial interactions between species and food. An integral spoilage process involved interactions of bacteria to bacteria, metabolites to metabolites, bacteria to metabolites, and bacteria to food matrix. Great differences in volatile organic compounds were observed between bacteria grown in standard culture and bacteria grown in the meat matrix, which further verified the viewpoint that the odor of bacteria obtained from growth culture cannot represent and characterize the spoilage process of certain food caused by the corresponding bacteria.

## Conclusion

In this study, not only has a novel MAP approach been developed for extending 4-day shelf life of yellow-feathered broiler compared to PAP, but the dynamics of microbial diversity were also characterized. Bacterial community shift was dramatically affected by packaging patterns in chilled broiler carcasses, and the species of dominant bacteria were increased in MAP, with top spoilage genera of *Aeromonas*, *Lactococcus*, *Serratia*, and *Shewanella*, whereas only *Pseudomonas* became the dominant bacteria of broiler carcass under penetrated-air packaging pattern. Meanwhile, the dominant spoilage strains displayed strong proteolytic activity, and volatile organic compounds of spoilage meat was varied with that of each representative strain grown in standard culture. Although our data have enabled new insights into community composition of yellow-feathered broiler meat during spoilage process, more studies on dominant species are expected to be done in the future to (1) confirm the potential link of off-odors between natural meat spoilage and dominant species metabolism on meat *in situ*, and (2) illustrate the unique gene pathways of dominant species contribution to slime, discolor, and texture softening of yellow-feathered broiler meat.

## Author Contributions

HW and XX designed research; HW, XZ, GW, and KJ performed research; HW, XZ, and GZ analyzed data; HW and XX wrote the paper.

## Conflict of Interest Statement

The authors declare that the research was conducted in the absence of any commercial or financial relationships that could be construed as a potential conflict of interest.
