# Supplementary material for: Bacterial Community and Spoilage Profiles Shift in Response to Packaging in Yellow-Feather Broiler, a Highly Popular Meat in Asia
Source: Front Microbiol. 2017 Dec 22;8:2588. doi: 10.3389/fmicb.2017.02588 (PMC5743932; doi:10.3389/fmicb.2017.02588)
Supplement: Supplementary file 1 [file Table_1.DOCX]

Table S1 Overview of the data processing and assembly analysis of metagenomic sequence

| Data pre-processing | Statistics | Assembly analysis | Statistics |
| --- | --- | --- | --- |
| Total raw data (Mbp) | 56,642.68 | Scaffolds (average) | 72,213 |
| Average raw data (Mbp) | 6,293.63 | Total length (bp) | 820,974,610 |
| Total clean data (Mbp) | 56,524.61 | N50 length (bp) | 1,240.20 |
| Average clean data (Mbp) | 6,280.51 | N90 length (bp) | 583.20 |
| Effective percent (%) | 99.79 | Scaftigs (average) | 70,604 |
| Total no-host data (Mbp) | 51,991.58 | Total length (bp) | 775,303,201 |
| Average no-host data (Mbp) | 5,776.84 | N50 length (bp) | 1,173 |
| Effective rate (%) | 91.98 | N90 length (bp) | 577 |

Table S2 Overview of prediction, annotation and taxonomy of genes

| Genes prediction | Statistics | Annotation and taxonomy | Statistics |
| --- | --- | --- | --- |
| Total ORFs | 1,171,588 | Phylum level (%) | 97.58 |
| Average ORFs | 117,159 | Class level (%) | 96.25 |
| Gene catalogue | 403,555 | Order level (%) | 93.73 |
| Complete ORFs number | 51,663 | Family level (%) | 92.57 |
| Total length (Mbp) | 258 | Genus level (%) | 84.00 |
| Average length (bp) | 639 | Species level (%) | 49.67 |
| GC percent (%) | 52.50 | Unclassified (%) | 1.12 |


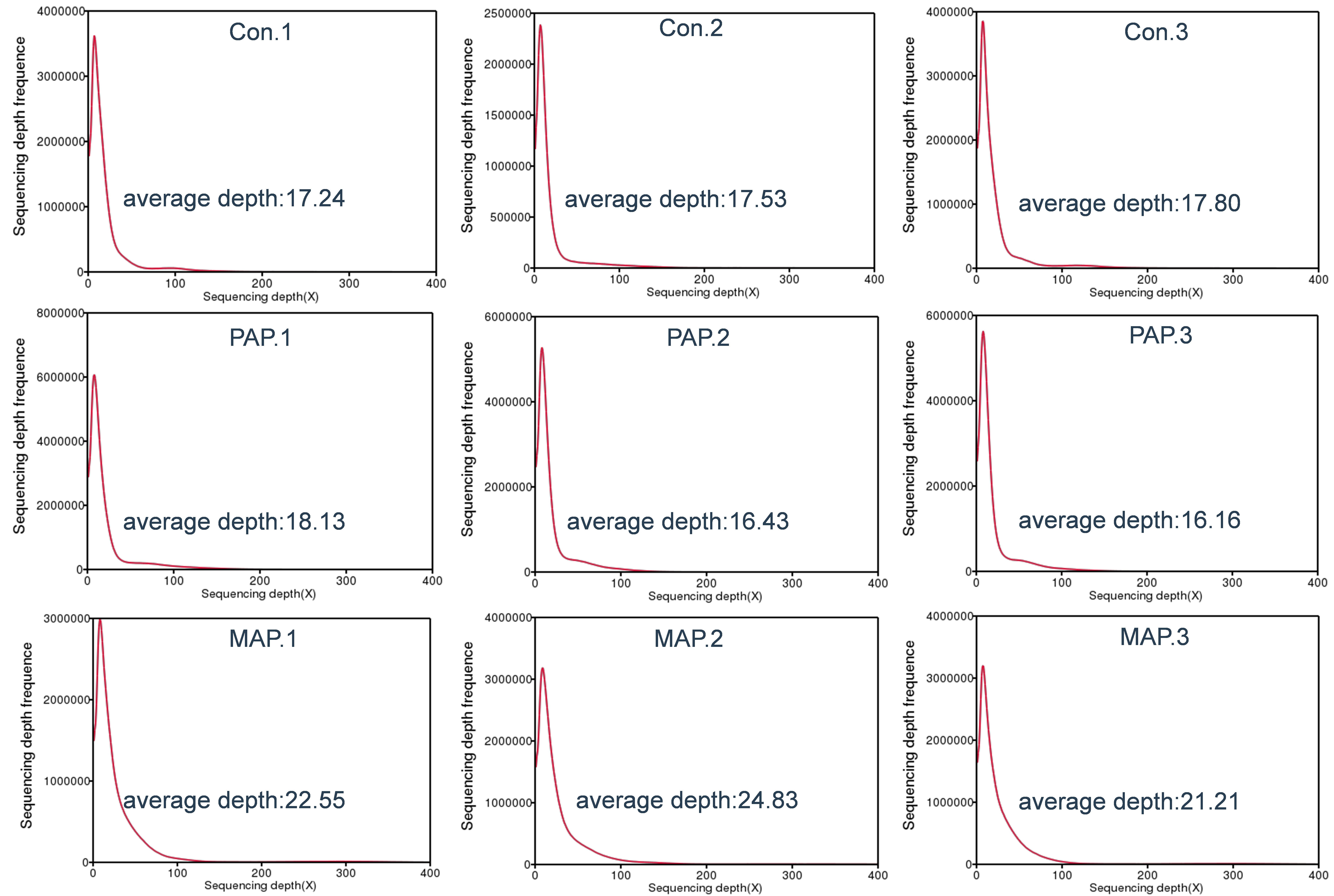


Figure S1 The sequencing depth of metagenomics in each sample
